# Behavioral Health as a Key Component of Integrated Care for Dually Eligible Medicare/Medicaid Beneficiaries

**DOI:** 10.1093/geroni/igaf122.2700

**Published:** 2025-12-31

**Authors:** Pamela Nadash, Janelle Fassi, Elizabeth Simpson, Calvin Tran, Marc Cohen

**Affiliations:** University of Massachusetts Boston, Boston, Massachusetts, United States; University of Massachusetts Boston, Boston, Massachusetts, United States; University of Massachusetts Boston, Boston, Massachusetts, United States; University of Massachusetts Boston, Boston, Massachusetts, United States; University of Massachusetts Boston, Boston, Massachusetts, United States

## Abstract

The integration of behavioral health into managed care programs for dually eligible individuals is understudied. Behavioral health has traditionally been siloed into separate managed care plans: only recently has policy encouraged a shift toward integration. This presentation discusses findings from a qualitative study of an innovative managed care plan, Commonwealth Care Alliance, which serves younger and older dually eligible members in separate managed care plans. Each plan enrolls high proportions of individuals with serious and persistent mental illness (SPMI): in the program serving people 18-65, 52% have an SPMI diagnosis and 35% have SUD (substance use disorder). In the older adult program, 19% have an SPMI diagnosis and 13% have SUD. Using data from 22 key informants (primarily program staff) and 41 plan members, this study analyzes program elements specific to behavioral health and member experiences. It finds behavioral health expertise woven throughout the program, along with a holistic care focus: most importantly, members with SPMI/SUD are likely to have specialized behavioral health care coordination. The plan works closely with community-based mental health providers, which operate as health homes for some members; the plan also utilizes crisis stabilization units to reduce admissions to psychiatric hospitals and integrated mobile health for crisis interventions. Members report high levels of satisfaction with their access to and quality of behavioral healthcare providers, and appreciate the plans’ support for food, housing, and transportation, which enables consistent participation in treatment. Reported dissatisfaction stems from poor communication with plans, particularly due to high staff turnover.

